# The effect of self-compassion versus mindfulness interventions on autonomic responses to stress in generalized anxiety disorders

**DOI:** 10.3389/fpsyt.2025.1483827

**Published:** 2025-02-04

**Authors:** Xuejun Qi, Yonghui Shen, Xianwei Che, Ying Wang, Xi Luo, Lijun Sun

**Affiliations:** ^1^ Affiliated Mental Health Center & Hangzhou Seventh People’s Hospital, Zhejiang University School of Medicine, Hangzhou, China; ^2^ Centre for Cognition and Brain Disorders, The Affiliated Hospital of Hangzhou Normal University, Hangzhou, China; ^3^ School of Nursing, Hangzhou Medical College, Hangzhou, China

**Keywords:** mindfulness, self-compassion, heart rate, generalized anxiety disorder, stress

## Abstract

**Objective:**

Although research on psychological interventions in generalized anxiety disorder (GAD) has provided evidence of their effectiveness regarding self-reported outcomes, few studies have examined their psychophysiological effects. Heart rate is emerging as a potential biomarker of efficacy in anxiety disorders. This study aimed to investigate the effects of a self-compassion intervention versus a mindfulness intervention on physiological arousal in response to induced stress.

**Methods:**

Forty-seven patients with GAD had heart rate data collected during a stress task before and after a 2-week pharmacological treatment (known as treatment as usual, TAU), a self-compassion intervention + TAU or a mindfulness intervention + TAU. They also reported state anxiety, positive affect, and negative affect at pre- and post- intervention before the stress task. ANOVAs were conducted to analyze the effects on electrocardiogram data self-reported measurements.

**Results:**

Self-compassion intervention uniquely decreased heart rate response to a stressor whereas mindfulness intervention did not. Both treatments decreased state anxiety and negative affect to a stressor, while increased positive affect in this context. We also demonstrated a significant correlation between decreased heart rate response and less negative emotions.

**Conclusion:**

The Findings provides novel physiological evidence that self-compassion interventions buffer stress reactivity in individuals with GAD. Attention shall be paid to the limitations in small and unequal sample size and a non-randomized study design.

## Introduction

Generalized anxiety disorder (GAD) is characterized by persistent worrying, tension, anxiety and other somatic symptoms (1, 2). GAD is one of the least successfully treated anxiety disorder with drugs or psychotherapies (3, 4). To this end, certain cognitive behavioral and mindfulness-based therapies are being developed in the past few decades for GAD. Among them, self-compassion and mindfulness interventions are potent psychological therapeutics for GAD (5–9).

Self-compassion, defined as being supportive of oneself during experiences of distress or pain, has consistently been shown to promote mental health and reduce anxiety and depression (10–12). Indeed, our recent trial and meta-analyses have both confirmed the benefits of self-compassion interventions for GAD individuals (8, 9). However, fewer studies have examined the effects of self-compassion interventions on sympathetic arousal in GAD populations. Beyond GAD, self-compassion is suggested to have a soothing effect on sympathetic arousal to a stressor, such as heart rate (13–15), which is used to indicate autonomic nervous system activation in anxiety disorders (16, 17). However, there is a paucity of evidence to support this benefit in GAD individuals.

As another potent therapy, mindfulness interventions cultivate moment-to-moment awareness in a non-judgmental and accepting manner, which have shown promise as effective treatments for GAD in recent years (5, 7, 18). Mindfulness is suggested to improve emotion regulation and relaxation, and thus is likely to lower sympathetic arousal (19, 20). However previous studies have demonstrated mixed findings on the effects of mindfulness interventions on physiological arousal (21–23). For instance, one study found that mindfulness interventions produced a significant reduction in heart rate to stressful experiences (24). In contrast, another study found mindfulness trainings to have no effect on physiological arousal to negative experiences, such as heart rate and blood pressure (25, 26). Together with self-compassion and mindfulness interventions, overall, there is a necessity to clarify the treatment effect on sympathetic arousal in individual with GAD.

According to Neff’s theory, mindfulness is a core component of self-compassion (27), yet self-compassion and mindfulness appear to engage distinct physiological systems. Mindfulness has been associated with increased activity in the middle prefrontal brain regions, whereas compassion is linked to the mammalian caregiving system (28, 29). Some studies suggest that self-compassion is a stronger predictor of well-being than mindfulness, although findings related to anxiety are mixed (18, 30–32). Few studies have directly compared self-compassion and mindfulness interventions in the GAD population. Therefore, clinical trials that directly compare these interventions are needed to validate previous findings and provide additional confirmation of biological effects to understand the overlapping and unique benefits of each.

This study was embedded within a non-randomized clinical trial that evaluated the effects of a self-compassion intervention and a mindfulness intervention compared to treatment as usual (TAU) in a sample of patients with GAD (9). The current study examined the effects of a mindfulness intervention and a self-compassion intervention on heart rate in response to induced stress. It is noted that heart rate was used to index sympathetic arousal to a stressor in this context, whereby heart rate variability was not used due to its requirement of longer duration of data for analysis (33, 34). We were also interested in whether the mindfulness intervention and the self-compassion intervention would improve mood, since meditation and self-compassion practices are linked to increased positive mood (35–37). We hypothesized that both the self-compassion and mindfulness groups would exhibit decreased heart rate in response to induced stress after the intervention. We also expected that both interventions would reduce state anxiety and negative affect.

## Method

### Participants and procedure

This is a *post-hoc* study using data from a nonrandomized controlled trial (9) assessing a self-compassion intervention and a mindfulness training compared to TAU in adult patients diagnosed with GAD. We recruited individuals with GAD symptoms to participate in the study in the Hangzhou Seventh People’s Hospital through advertisement posters and flyers. Trained clinicians conducted a DSM-5 principal diagnostic evaluation of GAD using Mini-International Neuropsychiatric Interview (M.I.N.I.) [American Psychiatric (38, 39)]. Inclusion criteria were adults aged 18 to 65 with GAD, Hamilton Anxiety Rating Scale ≥ 14, Hamilton Depression Rating Scale < 23. Exclusion criteria included psychiatric and medical comorbidities, such as bipolar disorder, suicidal ideation or risk, alcohol or substance use disorder, severe physical disease, cognitive dysfunction or hearing impairment, currently other psychotherapy. All participants gave informed written consent before beginning the study (Ethics committee in the Hangzhou Seventh People’s Hospital 2021067).

**Figure 1:**
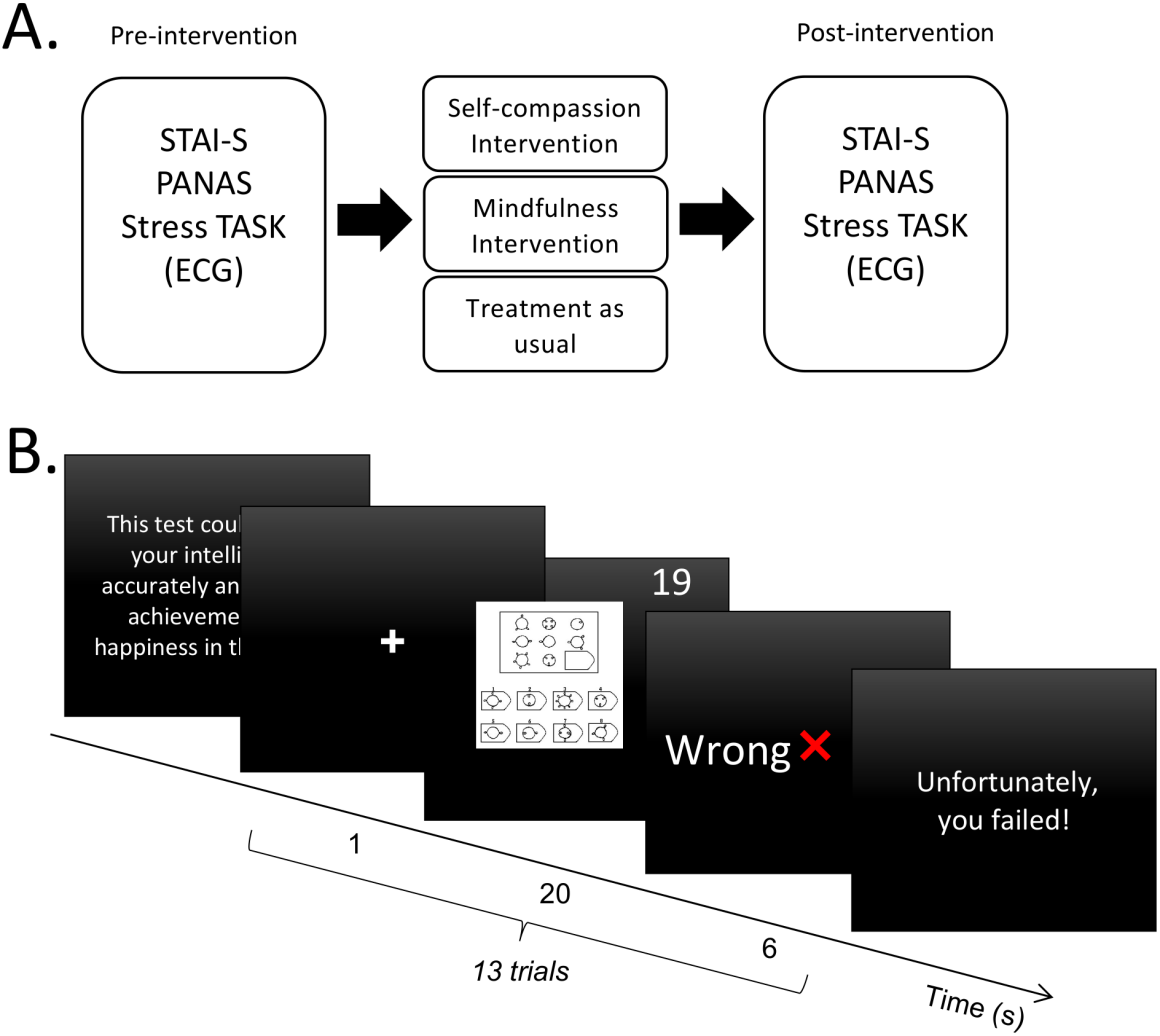
Study design and procedure. **(A)** Experimental procedure of this study. **(B)** Schematic diagram of the Stress Task. A total of 13 trials were performed. STAI-S, State form of Spielberger’s State-Trait Anxiety Inventory; PANAS, Positive and Negative Affect Schedule; ECG, electrocardiograph.

Patients in the Self-compassion group and the Mindfulness group received eight intervention sessions in two weeks in addition to usual treatment (i.e., pharmacotherapy). See Luo, Shen (9) for more details. After the clinical interview and baseline questionnaire measurements, participants were set up for the electrocardiograph (ECG) recording and then underwent a Stress Task (40, 41). At the end of the intervention, they completed questionnaires and the second Stress Task with ECG recording (**Figure 1A**).

Seventy-five patients participated in the parent study with 25 in each group (9). In the present study, 47 participants (Self-compassion group = 19; Mindfulness group = 17; TAU = 11) were analyzed with complete heart rate data in the Stress Task at both pre- and post-intervention. The excluded participants (n = 28) either had a lack of post-intervention data (8 Self-compassion group, 8 TAU group), technical issues (2 Self-compassion group, 2 Mindfulness group, 2 TAU group), or constant muscle noise (3 Mindfulness group, 3 TAU group).

### The stress task

Same as the previous studies, 13 difficult items in the Raven Standard Reasoning Test (Chinese City Edition) were selected, i.e., B12, C10, C12, D9, D10, D11, D12, E7, E8, E9, E10, E11, E12 (40, 41). At the beginning of the Stress Task, participants were told that the test could accurately assess intelligence and predict future achievement and happiness. The task consisted of 13 trials. Each trial began with a one-second fixation, followed by a reasoning item with a 20-second countdown during which participants were asked to think and respond. After the countdown, the computer displayed feedback on their answers for six seconds. Two questions were randomly marked ‘Correct’, and the rest were marked ‘Wrong’ as negative feedback. At the end of the task, the screen displayed the message: ‘Unfortunately, you failed!’ (**Figure 1B**).

### Interventions

Both the self-compassion intervention and mindfulness intervention are group-based interventions with eight sessions over two weeks. Patients in the two active groups received interventions in addition to pharmacological treatments. Specifically, in the self-compassion group, various practices such as affectionate breathing, stand with compassion, compassionate body scan, compassionate movement, self-compassion breaks, self-compassion meditation for ourselves are employed to develop the ability to comfort oneself during periods of distress. The mindfulness intervention was designed to use body movement as an anchor, and included mindful breathing, standing, stretching, as well as mindful awareness of sounds and thoughts, in order to cultivate awareness of present-moment internal experiences with acceptance and nonjudgment. In the TAU group, patients only received the pharmacotherapy (9).

### Measures

#### State form of Spielberger’s State-Trait Anxiety Inventory

This self-reported scale assesses state anxiety via 20 items on a 4-point Likert scale (1 = not at all to 4 = very much so) (42). The total score ranges from 20 to 80, with higher scores indicating greater anxiety. The Chinese version is well-validated (43).

#### Positive and Negative Affect Schedule

The PANAS is a commonly used self-reported measure containing two subscales: positive affect subscale and negative affect subscale. Each subscale includes 10 emotion words to assess positive or negative emotions (44). Participants responded according to how they felt over the last few days using a 5-point scale (1 = very slightly to 5 = most of the time). Higher scores indicate higher positive or negative affect. The PANAS has been validated for use with Chinese people (45).

#### Hamilton Anxiety Rating Scale

The HAMA is a well-validated and clinician-rated instrument designed to assess anxiety severity (46). It consists of 14 items, each scored from 0 (no symptoms) to 4 (severe symptoms). The total score ranges of 0 to 56. It is validated for the Chinese population (47).

#### Hamilton Depression Rating Scale

This clinician-rated scale evaluates depressive symptoms (48). It comprises 17 items, each rated from 0 (no symptoms) to 4 (the worst symptom severity), with a total score range from 0 to 52. The Chinese version has excellent interrater reliability and good validity (49).

#### Heart rate

Heart rate (HR) as an indicator of physiological arousal was continuously monitored using a BITalino (r)evolution Board Kit BT (BITalino, Portugal) (http://bitalino.com/en/). Three Ag/AgCI electrodes were placed on the chest, with two near the clavicles bilaterally and one at the lower edge of the left rib cage. Electrocardiogram data was recorded through the OpenSignals (r)evolution software (v.2017, BITalino, Portugal) at a 1000Hz sampling rate.

### Data analysis and statistics

Heart Rate data were preprocessed as previously described (50). Inter-beat-interval (IBI) series were derived using the Pan-Tompkins algorithm, which identifies the R wave peak as the fiducial point (51). Artifacts were visually inspected and edited if necessary according to published guidelines (52). IBI series were then converted to beat-per-minute (BPM) series. Continues data were segmented based on the onset of the feedback (-1 to 6 s). Trials with ‘Wrong’ feedback were retained and baseline corrected for each trial (-1 to 0 s, with 0 as the onset of the feedback) to control for individual baseline heart rate differences and capture the dynamics of event-related heart rate changes in a short period (53). We analyzed heart rate during the six seconds following negative feedback and averaged these data across trials for each participant. We then compared heart rate changes from baseline to post-intervention in each group. It is worth noting that heart rate was analyzed here instead of heart rate variability metrics in the time (e.g. root mean square of successive differences between heartbeats) (54) or frequency domain (e.g. high-frequency heart rate variability) (55). This was done as this study designed a stress-induced sympathetic arousal in a short time window (i.e. 6 sec) whereby heart rate variability requires longer duration of data for analysis (33). To capture treatment effects in a narrow window of sympathetic arousal, a sliding time window approach was adopted here, which is more sensitive to statistical differences in dynamic heart rate changes (56). Specifically, the step size was specified as 50ms and the window length as 500ms (56). The 6-sec window was then examined in each 500ms windows from 0 to 6 seconds. In each window, paired sample t-test was conducted to compare heart rate changes between pre- and post-intervention. These settings were standardized for each group, which could identify different significant time intervals related to different treatments.

An initial comparison of baseline demographic and clinical characteristics between groups was conducted using SPSS (version 23; IBM Corp, Armonk, NY) with independent sample t-tests for continuous variables and chi-square (χ²) tests for categorical variables. Two-way ANOVAs (intervention group: SC, Mindfulness, TAU; time: Pre, Post) were conducted for STAI-S and PANAS. *Post-hoc* pairwise comparisons were performed using a Bonferroni correction (α < 0.05). For heart rate data, baseline correction from pre- to post-treatment was initially performed for each treatment as there was a baseline difference across groups (*p* = 0.008). One-way ANOVA (intervention group: SC, Mindfulness, TAU; time: Pre, Post) was then conducted for heart rate change data (6-sec average). *Post-hoc* pairwise comparisons were performed using a Bonferroni correction (α < 0.05). Additionally, Pearson correlation analyses were performed to examine the relationships between heart rate change scores and subjective measurements.

## Results

### Demographic and descriptive analysis

Demographic characteristics are presented in **Table 1**. There were no significant differences in age, gender, education, employment, anxiety as well as depression level (*p*_s_ > 0.05). There was significance in marital status (*p* = 0.046).

**Table 1:** Baseline Characteristics of Patients.

	Total (*n* = 47)	SC Group (*n* = 19)	Mindfulness Group (*n* = 17)	TAU Group (*n* = 11)	*p* value^a^
Age, years: mean (SD)	39.87(12.25)	43.37(11.62)	40.71(10.81)	32.55(13.32)	0.06
Gender, n (%)					
Female Male	28(59.60) 19(40.40)	10(52.60) 9(47.40)	10(58.80) 7(41.20)	8(72.70) 3(27.30)	0.54
Marital Status, n (%)					
Married Single/Separated	32(68.10) 15(31.90)	15(78.90) 4(21.10)	13(76.50) 4(23.50)	4(36.40) 7(63.60)	0.046
Education, n (%)					
Secondary High school University degree Postgraduate degree	12(25.50) 12(25.50) 19(40.40) 4(8.50)	6(31.60) 5(26.30) 6(31.60) 2(10.50)	4(23.50) 6(35.30) 5(29.40) 2(11.80)	2(18.20) 1(9.10) 8(72.70) 0(0)	0.56
Employment, n (%)					
Unemployed/Housewife/Retired Employed	17(36.20) 30(63.80)	8(42.10) 11(57.90)	5(29.40) 12(70.60)	4(36.40) 7(63.60)	0.64
STAI-S score, mean (SD)	46.26(10.82)	47.37(7.19)	42.06(12.47)	50.82(11.91)	0.09
PANAS-PA score, mean (SD)	24.36(6.14)	24.89(4.95)	24.35(8.42)	23.45(3.78)	0.83
PANAS-NA score, mean (SD)	29.70(7.47)	30.63(6.73)	26.53(8.06)	33.00(6.34)	0.06
HAMA score, mean (SD)	21.38(5.17)	19.84(3.56)	22.88(6.57)	21.73(4.78)	0.21
HAMD score, mean (SD)	14.43(4.72)	12.79(3.61)	15.53(5.27)	15.55(5.11)	0.15

SC group, Self-compassion Group; TAU group, Treatment as usual Group; STAI-S, State form of Spielberger’s State-Trait Anxiety Inventory; PANAS-PA, Positive and Negative Affect Schedule – Positive subscale; PANAS-NA, Positive and Negative Affect Schedule – Negative subscale; HAMA, Hamilton Anxiety Rating Scale; HAMD, Hamilton Depression Rating Scale.

a: Estimated by χ2 test for categorical variables, and ANOVA for continuous variables.

### State anxiety and PANAS

**Figure 2:**
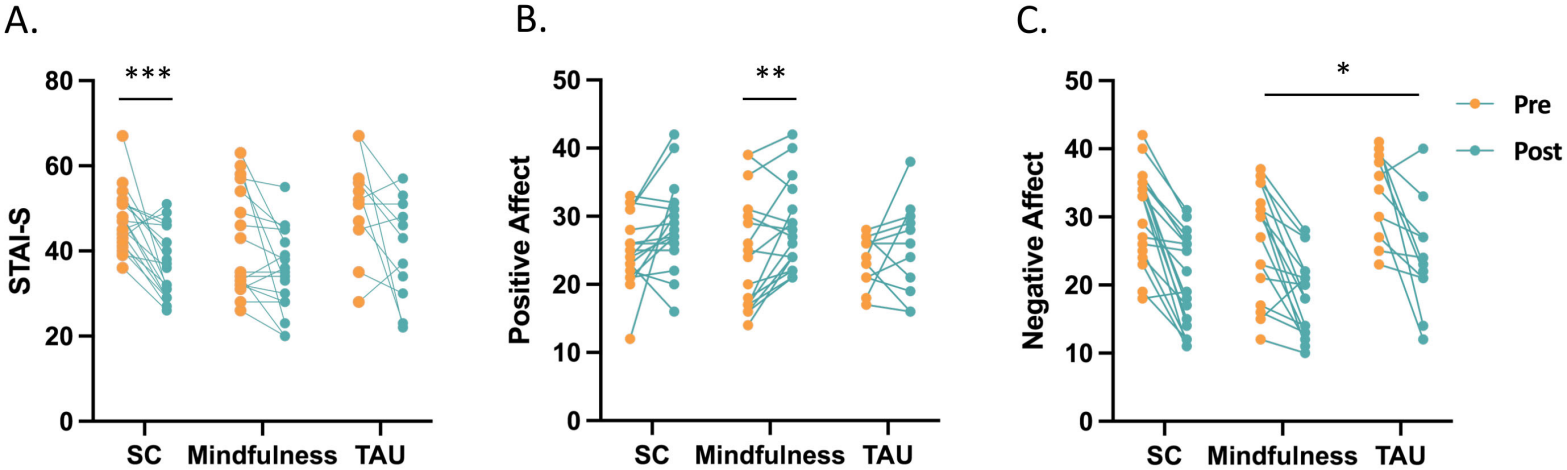
State anxiety and affect responses in the Self-compassion (n = 19), Mindfulness (n = 17), and Treatment as usual (n = 11) group. All groups showed a main effect of time: **(A)** The time effect on state anxiety was driven by changes in the Self-compassion group (*t* = 4.65, *p_corrected_* < 0.001). **(B)** The time effect on positive affect was driven by changes in the Mindfulness condition (*t* = - 3.07, *p* = 0.007). **(C)** *Post-hoc* analysis of a group effect revealed a significant difference in negative affect between the Mindfulness group and the TAU group. STAI-S, State form of Spielberger’s State-Trait Anxiety Inventory; PANAS-PA, Positive and Negative Affect Schedule – Positive subscale; PANAS-NA, Positive and Negative Affect Schedule – Negative subscale; SC, Self-compassion group; Mindfulness, Mindfulness group; TAU, Treatment as usual Group. * *p* < 0.05, ** *p* < 0.01, *** *p* < 0.001.

Due to small sample sizes, the sphericity assumption is violated for most of our variables (*p_s_* > 0.05). Data were therefore reported with the Greenhouse–Geisser correction (**Table 2**). For state anxiety, a two-way ANOVA revealed a main effect of time (F_1,44_ = 26.61, *p* < 0.001, $\eta_{p}^{2}$ = 0.38), suggesting that state anxiety decreased significantly (*p_corrected_* < 0.001) from pre-intervention (*Mean* = 46.75) to post-intervention (*Mean* = 37.41) across the three groups. Further analysis showed that this time effect was mainly driven by the changes in the Self-compassion group (*t* = 4.65, *p_corrected_* < 0.001) (**Figure 2A**).

**Table 2:** ANOVA results for effects of intervention on state anxiety, affect responses and heart rate change.

Effect	Sum of Squares	*df*	Mean Square	*F*	*p*	* $\eta_{p}^{2}$ *
*State anxiety*						
Time	1940.89	1	1940.89	26.61	0.00	0.38
Group	666.52	2	333.26	2.55	0.09	0.10
Time &Group	71.42	2	35.71	0.49	0.62	0.02
*Positive affect*						
Time	205.58	1	205.58	11.20	0.002	0.20
Group	75.68	2	37.84	0.61	0.55	0.03
Time &Group	14.92	2	7.46	0,41	0,67	0.02
*Negative affect*						
Time	1868.33	1	1868.33	67.36	0.00	0.61
Group	506.08	2	253.04	3.66	0.034	0.14
Time &Group	18.84	2	9.42	0.34	0.71	0,02
*Heart rate change*						
Group	55.43	2	27.71	5.35	0.008	

For positive affect, a two-way ANOVA also revealed a main effect of time (F_1,44_ = 11.20, *p* < 0.01, $\eta_{p}^{2}$ = 0.20), suggesting that positive affect increased significantly (*p_corrected_* < 0.001) from pre-intervention (*Mean* = 24.23) to post-intervention (*Mean* = 27.28) in all three groups. Further analysis indicated that this time effect was mainly driven by the changes in the Mindfulness group (*t* = -3.07, *p* = 0.007) (**Figure 2B**).

For negative affect, a two-way ANOVA revealed a main effect of time (F_1,44_ = 67.36, *p* < 0.001, $\eta_{p}^{2}$ = 0.61) and a group effect (F_2,44_ = 3.66, *p* = 0.034, $\eta_{p}^{2}$ = 0.14). The time effect suggested that negative affect reduced significantly (*p_corrected_* < 0.001) from pre-intervention (*Mean* = 30.05) to post-intervention (*Mean* = 20.89) in all groups. In terms of the group effect, according to *post-hoc* analysis, a significant difference was found between the Mindfulness group and TAU group (*p_corrected_* = 0.031, **Figure 2C**).

### Heart rate change

**Figure 3:**
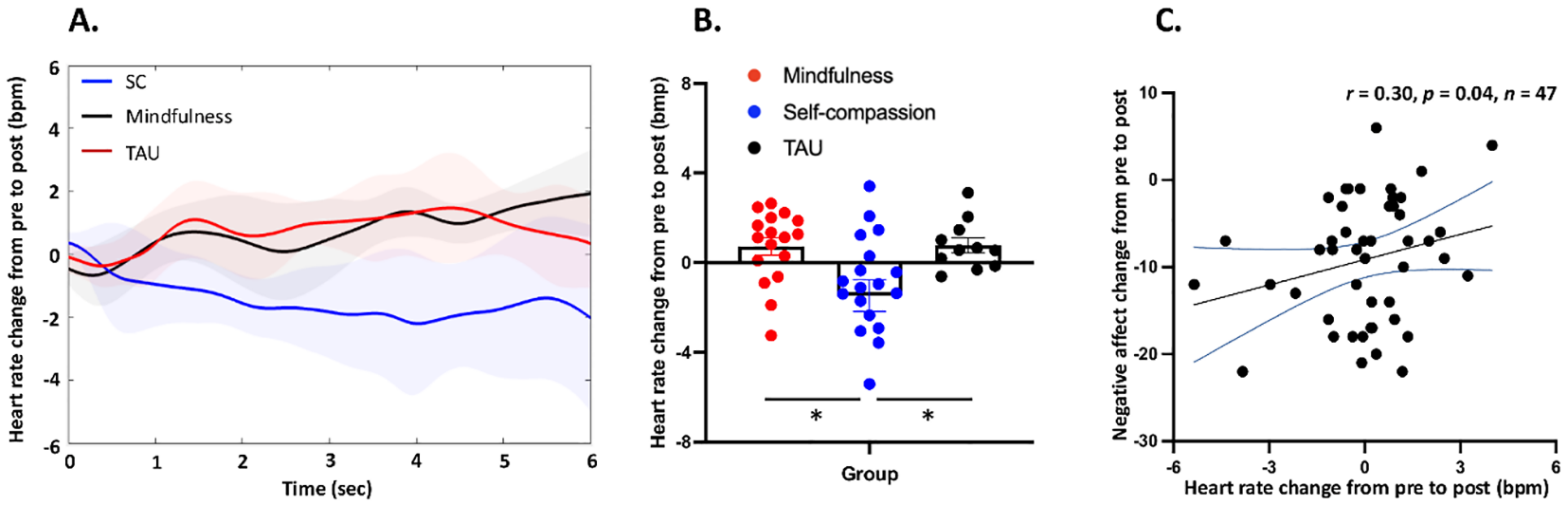
Heart rate results after the negative feedback in the Self-compassion (n = 19), Mindfulness (n = 17), and Treatment as usual (n = 11) group. **(A)** Heart rate dynamics across groups and time. **(B)** Self-compassion treatment reduced heart rate response than both the mindfulness (*p_corrected_* = 0.018) and TAU treatment (*p_corrected_* = 0.037). Meanwhile, no significant difference in heart rate change was found between the mindfulness and the TAU group (*p_corrected_* = 1.000). **(C)** In all three groups, heart rate changes from pre- to post-treatment were negatively associated with changes in negative affect (*r* = 0.30, *p* = 0.04, *n* = 47). SC, Self-compassion Group; Mindfulness, Mindfulness Group; TAU, Treatment as usual Group.

One-way ANOVA revealed a main effect of group (F_2,46_ = 5.35, *p* = 0.008). *Post-hoc* tests indicated that self-compassion treatment reduced heart rate response than both the mindfulness (*p_corrected_* = 0.018) and TAU treatment (*p_corrected_* = 0.037). No significant difference in heart rate change was found between the mindfulness and the TAU group (*p_corrected_* = 1.000) (**Figures 3A, B**).

### Correlation analyses

When data were pooled across the three groups, decreased heart rate from pre- to post-intervention was associated with less negative emotions (*r* = 0.30, *p* = 0.04, *n* = 47). No other significant correlations were identified (**Figure 3C**).

## Discussion

The aim of this study was to examine the effects of self-compassion and mindfulness intervention on the sympathetic stress response in patients with GAD. Overall, self-compassion intervention uniquely decreased heart rate response to a stressor whereas mindfulness intervention did not. Both treatments decreased state anxiety and negative affect to a stressor, while increased positive affect in this context. We also demonstrated a significant correlation between decreased heart rate response and less negative emotions.

Although recent studies have confirmed the benefits of self-compassion interventions for GAD individuals (8, 9), it is largely unknow their impact on sympathetic arousal in GAD populations. Using a stressor induction paradigm within a pre- to post-treatment design, we provided novel evidence that a 2-week self-compassion intervention significantly decreased heart rate response to a stressor. Heart rate has been used to indicate sympathetic arousal in anxiety disorders (16, 17). Our data suggested that self-compassion plays a crucial role in downregulating the autonomic nervous system and regulating emotions in the context of GAD (8, 57, 58). Our clinical data further corroborate this finding, in which self-compassion intervention decreased anxiety but increased positive affect. These findings are consistent with previous views that self-compassion is able to activate the soothing and caring system, which is characterized by a calm and reduced physiological arousal (13, 59, 60).

Interestingly, mindfulness intervention had no effect on heart rate response to a stressor, which is inconsistent with previous research (24, 60). This is rather uncommon in stress experiments, but could be explained by the clinical population with low flexibility of the autonomic nervous system (61, 62). Many GAD patients have suffered from this intractable and refractory disease for years. They might not experience a reduction in physiological arousal after short-term interventions. In addition, a dose-response effect may also play a role in this null effect, whereby a 2-week mindfulness intervention is not enough to modulate physiological response in a clinical sample, especially in the context of a stressor induction task (63, 64). However, it is noted that there is mixed evidence on the effects of mindfulness intervention on sympathetic arousal (24, 26). It is possible that differences in mindfulness interventions may have played a role in this inconsistency. Future studies are therefore needed to clarify the impact of treatment duration, course content and designs in this context.

The different physiological reactivity observed between the two treatments suggests that these practices may involve distinct mechanisms (65). While direct comparisons are limited in terms of the physiology effects of self-compassion versus mindfulness, researchers have proposed that compassion is associated with mammalian caregiving systems. This involves oxytocin and other hormones related to feeling of attachment and safety, as well as brain activity related to love and affiliation (66, 67). In contrast, mindfulness has been linked to brain activity in the middle prefrontal regions, representing a relatively recent evolutionary development (28). This mechanistic difference may be associated with the distinct effects on sympathetic arousal to a stressor in the current study. These findings could also be considered in the context of HRV. Previous researches have shown that both self-compassion and mindfulness are associated increased HRV (57, 68–70). Therefore, self-compassion and mindfulness may exert distinct effects on the sympathetic and parasympathetic nervous systems.

We have also provided interesting findings that these two treatments have unique advantages over distinct aspects of emotions. Specifically, self-compassion intervention is more effective in reducing state anxiety whereas the mindfulness treatment has an advantage in regulating positive and negative affect. These findings are novel as they specified different aspects of emotions that self-compassion and mindfulness interventions could better target. They also provide insights on the contexts for a certain treatment in clinical practices.

Although we have presented interesting findings, they should be treated with caution. First, conclusions are limited due to relatively small sample size, and more patients should be recruited for future studies. Another limitation is that the study was not randomized due to limited space and therapist, although no differences were found in most baseline characteristics. There were some other limitations in this study. Although heart rate variability (HRV) is another common marker of psychophysiological stress, it typically requires a longer duration of data for analysis (33, 34). With only six seconds, we chose HR to analyze our data. In addition, although the stress task adopted in this study is simple and easy to conduct in clinical settings, using a classical stress task paradigm, such as the Trier Social Stress Test, would be more conducive to the generalization of research results.

Our results may have clinical implications. Self-compassion interventions predict flexible physiological responses to stress, have great potential in helping emotion regulation and physiological adjustment in anxiety disorder patients. In fact, compassion-focused therapy is becoming prevalent in clinical practice (71). Furthermore, cultivating self-compassion could protect both mental and cardiovascular health by decreasing heart rate and sympathetic activation, which are risk markers for hypertension and cardiac events.

In conclusion, we provided novel evidence that self-compassion intervention may be an effective strategy to decrease physiology stress reactivity and improve state anxiety in patients with GAD. Attention shall be paid to the limitations in small and unequal sample size and a non-randomized study design. Future works are needed to further establish these novel findings in large and randomized studies.

## Data Availability

The datasets presented in this study can be found in online repositories. The names of the repository/repositories and accession number(s) can be found in the article/**Supplementary Material**.
