# Supplementary material for: The effect of self-compassion versus mindfulness interventions on autonomic responses to stress in generalized anxiety disorders
Source: Front Psychiatry. 2025 Feb 4;16:1483827. doi: 10.3389/fpsyt.2025.1483827 (PMC11833619; doi:10.3389/fpsyt.2025.1483827)
Supplement: Supplementary file 1 [file Table1.docx]

**Table S1.** Correlation coefficients of variables in the Mindfulness group

| Variables | 1 | 2 | 3 | 4 | 5 | 6 | 7 | 8 | 9 | 10 | 11 | 12 | 13 | 14 | 15 | 16 |
| --- | --- | --- | --- | --- | --- | --- | --- | --- | --- | --- | --- | --- | --- | --- | --- | --- |
| 1. changes in HR (4.8-6s) | - |  |  |  |  |  |  |  |  |  |  |  |  |  |  |  |
| 2. HAMA_Pre | -0.41 | - |  |  |  |  |  |  |  |  |  |  |  |  |  |  |
| 3. HAMA_Post | -0.63** | 0.81** | - |  |  |  |  |  |  |  |  |  |  |  |  |  |
| 4. changes in HAMA (Post-pre) | 0.17 | -0.91** | -0.50* | - |  |  |  |  |  |  |  |  |  |  |  |  |
| 5. HAMD_Pre | -0.35 | 0.84** | 0.74** | -0.72** | - |  |  |  |  |  |  |  |  |  |  |  |
| 6. HAMD_Post | -0.35 | 0.46 | 0.65** | -0.22 | 0.59* | - |  |  |  |  |  |  |  |  |  |  |
| 7. changes in HAMD (Post-pre) | 0.13 | -0.65** | -0.36 | 0.70** | -0.73** | 0.12 | - |  |  |  |  |  |  |  |  |  |
| 8.STAI-S_Pre | -0.48 | 0.64** | 0.62** | -0.52* | 0.53* | 0.53* | -0.21 | - |  |  |  |  |  |  |  |  |
| 9. STAI-S_Post | -0.22 | 0.48 | 0.61** | -0.28 | 0.39 | 0.64** | 0.06 | 0.50* | - |  |  |  |  |  |  |  |
| 10. changes in STAI-S (Post-pre) | -0.34 | -0.30 | -0.15 | 0.34 | -0.25 | -0.03 | 0.28 | -0.67** | 0.31 | - |  |  |  |  |  |  |
| 11.PANAS-PA_Pre | 0.13 | -0.69** | -0.46 | 0.69** | -0.57* | -0.48* | 0.29 | -0.35 | -0.57* | -0.11 | - |  |  |  |  |  |
| 12. PANAS-PA_Post | 0.12 | -0.70** | -0.67** | 0.57* | -0.57* | -0.52* | 0.26 | -0.54* | -0.77** | -0.07 | 0.79** | - |  |  |  |  |
| 13. changes in PANAS-PA (Post-pre) | -0.05 | 0.24 | -0.09 | -0.41 | 0.21 | 0.13 | -0.14 | -0.12 | -0.04 | 0.09 | -0.64** | -0.02 | - |  |  |  |
| 14.PANAS-NA_Pre | -0.19 | 0.55* | 0.49* | -0.48 | 0.39 | 0.29 | -0.22 | 0.55* | 0.71** | 0.01 | -0.46 | -0.75** | -0.18 | - |  |  |
| 15. PANAS-NA_Post | -0.24 | 0.35 | 0.43 | -0.21 | 0.23 | 0.10 | -0.20 | 0.46 | 0.48 | -0.10 | -0.02 | -0.42 | -0.49* | 0.52* | - |  |
| 16. changes in PANAS-NA (Post-pre) | 0.17 | -0.34 | -0.20 | 0.37 | -0.24 | -0.25 | 0.09 | -0.25 | -0.42 | -0.09 | 0.51* | 0.50* | -0.19 | -0.71** | 0.24 | - |

*Note.* HR, Heart rate; HAMA, Hamilton Anxiety Rating Scale; HAMD, Hamilton Depression Rating Scale; STAI-S, State form of Spielberger’s State-Trait Anxiety Inventory; PANAS-PA, Positive and Negative Affect Schedule – Positive subscale; PANAS-NA, Positive and Negative Affect Schedule – Negative subscale.

n=17, **p* < 0.05, ***p* < 0.01.

**Table S2.** Correlation coefficients of variables in the Self-compassion group

| Variables | 1 | 2 | 3 | 4 | 5 | 6 | 7 | 8 | 9 | 10 | 11 | 12 | 13 | 14 | 15 | 16 |
| --- | --- | --- | --- | --- | --- | --- | --- | --- | --- | --- | --- | --- | --- | --- | --- | --- |
| 1. changes in HR (1.2-4.8s) | - |  |  |  |  |  |  |  |  |  |  |  |  |  |  |  |
| 2. HAMA_Pre | -0.05 | - |  |  |  |  |  |  |  |  |  |  |  |  |  |  |
| 3. HAMA_Post | -0.02 | 0.03 | - |  |  |  |  |  |  |  |  |  |  |  |  |  |
| 4. changes in HAMA (Post-pre) | 0.03 | -0.77** | 0.62** | - |  |  |  |  |  |  |  |  |  |  |  |  |
| 5. HAMD_Pre | -0.03 | 0.27 | 0.20 | -0.08 | - |  |  |  |  |  |  |  |  |  |  |  |
| 6. HAMD_Post | 0.17 | -0.34 | 0.68** | 0.70** | 0.20 | - |  |  |  |  |  |  |  |  |  |  |
| 7. changes in HAMD (Post-pre) | 0.11 | -0.43 | 0.15 | 0.43 | -0.88** | 0.30 | - |  |  |  |  |  |  |  |  |  |
| 8.STAI-S_Pre | 0.16 | 0.04 | -0.10 | -0.10 | 0.36 | 0.03 | -0.34 | - |  |  |  |  |  |  |  |  |
| 9. STAI-S_Post | 0.23 | -0.40 | 0.52* | 0.65** | -0.24 | 0.64** | 0.55* | 0.15 | - |  |  |  |  |  |  |  |
| 10. changes in STAI-S (Post-pre) | 0.07 | -0.35 | 0.49* | 0.60** | -0.45 | 0.49* | 0.69** | -0.60** | 0.70** | - |  |  |  |  |  |  |
| 11.PANAS-PA_Pre | -0.08 | -0.47* | 0.00 | 0.37 | -0.16 | 0.31 | 0.31 | -0.33 | 0.16 | 0.37 | - |  |  |  |  |  |
| 12. PANAS-PA_Post | 0.47* | -0.37 | -0.00 | 0.29 | -0.18 | 0.32 | 0.33 | -0.32 | -0.06 | 0.19 | 0.35 | - |  |  |  |  |
| 13. changes in PANAS-PA (Post-pre) | 0.51* | 0.01 | -0.00 | -0.01 | -0.05 | 0.08 | 0.08 | -0.06 | -0.18 | -0.11 | -0.43 | 0.70** | - |  |  |  |
| 14.PANAS-NA_Pre | -0.03 | 0.43 | 0.18 | -0.22 | 0.14 | 0.27 | 0.00 | 0.50* | 0.14 | -0.25 | -0.10 | -0.12 | -0.04 | - |  |  |
| 15. PANAS-NA_Post | 0.24 | 0.27 | 0.61** | 0.18 | 0.06 | 0.47* | 0.18 | -0.03 | 0.48* | 0.41 | 0.22 | 0.01 | -0.16 | 0.53* | - |  |
| 16. changes in PANAS-NA (Post-pre) | 0.28 | -0.17 | 0.45 | 0.42 | -0.08 | 0.21 | 0.18 | -0.55* | 0.36 | 0.68** | 0.34 | 0.14 | -0.13 | -0.47* | 0.50* | - |

*Note.* HR, Heart rate; HAMA, Hamilton Anxiety Rating Scale; HAMD, Hamilton Depression Rating Scale; STAI-S, State form of Spielberger’s State-Trait Anxiety Inventory; PANAS-PA, Positive and Negative Affect Schedule – Positive subscale; PANAS-NA, Positive and Negative Affect Schedule – Negative subscale.

n=19, **p* < 0.05, ***p* < 0.01.
